# The Coagulation Theory

**Published:** 1897-08

**Authors:** 


### THE COAGULATION THEORY.

The discussion upon this subject, which has taken a wide range and claimed the interest of many minds for years, seems to be drawing to a conclusion, if the very thorough experiments of Dr. E. L. York (*Dental Review*, July, 1897) are to be accepted. These investigations clearly prove that a coagulant, such as carbolic acid, does diffuse through dentine, notwithstanding assertions made to the contrary, and "does not form an impenetrable coagulum at the orificial ends of the dentinal tubuli." These investigations substantiate those previously made, and, conjoined with those presented in this issue by Dr. L. P. Bethel, form a series of facts that certainly settle the question.

It is a gratification, in this connection, to note that this work has been completed by two of the younger generation of dentists, demonstrating the value of the higher dental education adopted in all the dental colleges of the country. It is also satisfactory to observe that the younger men are not willing to accept age and experience as absolute authority, but seek for truth, where it alone can be found, in earnest and careful investigation.

